# Fatty Acid-Containing p(HEMA) Hydrogels; A Promising Coating Platform to Reduce Encrustation on Urinary Catheters

**DOI:** 10.3390/polym17040518

**Published:** 2025-02-17

**Authors:** David S. Jones, Gavin P. Andrews, Turlough Hamill, Brendan F. Gilmore

**Affiliations:** School of Pharmacy, Queen’s University of Belfast, 97, Lisburn Road, Belfast BT9 7BL, UK; g.andrews@qub.ac.uk (G.P.A.); b.gilmore@qub.ac.uk (B.F.G.)

**Keywords:** urinary catheters, p(HEMA), fatty acids, microbial adherence, reduced encrustation

## Abstract

Two significant clinical issues associated with the use of urinary catheters are catheter-associated urinary tract infection and encrustation. This study describes the design of novel hydrogels based on fatty acid-containing p(hydroxyethylmethacrylate, HEMA) and their resistance to both microbial adherence and encrustation. Incorporation of fatty acids increased the contact angle (surface hydrophobicity), decreased the ultimate tensile strength only after storage at pH 9 in artificial urine (AU) but not at lower pH values, decreased the Young’s modulus and % elongation at break (both stored in deionised water, AU pH 6 and AU pH 9) and decreased equilibrium swelling (only when stored in deionised water or AU pH 6 but not AU pH 9). Moderate reductions in adherence of *Escherichia coli*, *Proteus mirabilis* and *Staphylococcus epidermidis* to certain fatty acid containing (primarily decanoic acid and myristic acid) hydrogels were observed. No relationship was observed between hydrogel contact angle and resistance to microbial attachment. Most fatty acid-containing hydrogels exhibited significant, concentration-dependent resistance to encrustation, postulated to be due both to a greasy film resultant from the formation of the calcium/magnesium fatty acid salts at the surface and the role of Tween^®^ 80 in facilitating the removal of the fatty acid salts from the surface of the hydrogel. The observed enhanced resistance of the hydrogels to encrustation offers opportunities for the use of such systems as platforms for coatings of urinary catheters.

## 1. Introduction

Urinary catheters are one of the most prevalent indwelling medical devices, being primarily employed to ensure urine patency in acute and chronic disease settings [1,2,3]. Long-term urinary catheterisation refers to catheterisation for at least 28 consecutive days and is typically required for chronic conditions, including neurogenic bladder and prostatic hypertrophy [3,4]. Despite their widespread use, urinary catheters are principally associated with three clinical problems, namely catheter-associated urinary tract infection, encrustation and tissue trauma due to catheter insertion and removal, with a reported cost to the National Health Service in the UK between GBP 1 and 2.5 billion and circa 2500 deaths per annum [5]. It has been reported that the incidence of urinary tract infection is principally affected by the duration of catheterisation [3]. Catheter-Associated Urinary Tract Infection (CAUTI) is denoted by significant bacteriuria (≥10^5^ colony-forming units per mL of bacteria in a single urine sample and symptoms of infection [1]. Whilst not recommended, the use of antibiotics in patients who present with bacteriuria has been reported to delay microbial biofilm formation on catheters; however, there are subsequent implications with increased resistance of bacteria to antibiotics [6,7]. Furthermore, in patients who receive long-term hospital care, the majority of bloodstream infections have been reported to originate from CAUTI [1].

A second clinical concern with the use of urinary catheters and ureteral stents is encrustation, i.e., the deposition of insoluble salts (most frequently calcium and magnesium salts) onto the surface of urinary devices [8,9,10]. Whilst there may be a metabolic origin for this process, encrustation is most frequently associated with the presence of biofilms containing urease-producing pathogens, e.g., *Proteus mirabilis* and *Klebsiella pneumoniae*. The released urease catalyses the hydrolysis of urea to ammonia and carbon dioxide, resulting in an elevation of the pH of urine and the precipitation of insoluble calcium and magnesium phosphates [9]. The presence of encrustation on the surface of urinary devices is associated with several problems. Firstly, the crystalline nature of encrustation may cause damage to adjacent tissues when the device is removed. Secondly, the encrustation can protect the encased bacterial biofilm from physical removal and the action of antimicrobial agents and finally, encrustation may cause blockage of urinary flow, leading possibly to more serious consequences, e.g., pyelonephritis and sepsis [11].

There is a clinical need to develop urinary biomaterials that resist microbial biofilm formation and encrustation. Several approaches have been described. For example, biomaterials have been developed that offer a controlled release of antimicrobial agents, with a preference shown for non-antibiotic antimicrobial agents to minimise the development of resistance to antibiotics, e.g., silver [12,13,14], hexetidine [15] and chlorhexidine [16]. In contrast, modifications of the catheter surface have been described using, e.g., superhydrophobic coatings [11] and coatings, which undergo shedding as a function of pH [17]. Recently, Yao et al. described a coating for ureteral stents based on a clickable mussel-inspired antimicrobial peptide that offered significant antimicrobial activity and resistance to encrustation [18], whereas Yu et al. described a salt-triggered adaptive coating based on a chondroitin sulphate complex that offers antimicrobial and anti-encrustation properties [19].

The antimicrobial activity of fatty acids (primarily against Gram-positive microorganisms) has been known for many years [20]. Recently, Jin et al. reported the inhibitory properties of fatty acids (lauric acid, undecanoic acid and N-tridecanoic acid) against *Escherichia coli* biofilm (including problematic persister cells) [21]. Given the lack of resistance of microorganisms to the antimicrobial activity of fatty acids (in addition to their broad-spectrum antimicrobial activity), their use as antimicrobial additives for packaging materials has been described. For example, a coating composed of chitosan and lauric acid inhibited the formation of bacterial spoilage-derived volatile compounds, thereby enhancing the preservation of beef products [22]. Similarly, low-density polyethene films containing lauric acid exhibited antifungal activity and were proposed by the authors as promising packaging materials [23].

In this study, the formulation and characterisation of hydrogels based on poly(hydroxyethylmethacrylate), a commonly used urinary biomaterial containing a range of fatty acids (saturated and unsaturated) and the resistance of these films to microbial adherence and encrustation is reported for the first time. This study will, therefore, provide further insight into the potential use of these materials as urinary biomaterials with enhanced biological performance.

## 2. Materials and Methods

### 2.1. Chemicals

All chemicals were used as received and, unless otherwise stated, were of AnalaR grade or equivalent. Calcium chloride hexahydrate and magnesium chloride hexahydrate, both ≥97%, were purchased from VWR International, Leicestershire, UK. 2-Hydroxyethyl methacrylate (97%) (HEMA), ethylene glycol dimethacrylate (98%) (EGDMA), Urea, Chick ovalbumin, Grade II Crude dried egg white, potassium dihydrogen orthophosphate, urease (type IX from Jack Beans), urea, ethylene glycol dimethacrylate (EGDMA), 2,2′-Azo-bis(2-methylpropionitrile) (AIBN), Tween^®^ 80 (97%), caproic acid, decanoic acid, myristic acid, stearic acid (≥95%), oleic acid (90%), linoleic acid (≥95%) and sodium chloride were purchased from Sigma Aldrich, (Poole, Dorset, UK). Muller Hinton Agar (MHA) and Muller Hinton Broth (MHB) were purchased from Oxoid Limited (Basingstoke, UK).

### 2.2. Methods

#### 2.2.1. Preparation of Hydrogels

Hydrogels were prepared using 2-Hydroxyethyl methacrylate (HEMA), containing 2% (*w*/*w*) of the crosslinker ethylene glycol dimethacrylate (EGDMA) and the initiator *2,2*′-Azo-bis(2-methylpropionitrile) (AIBN) (0.5% *w*/*w*). Within these systems, a series of C_6_-C_18_ saturated and unsaturated fatty acids (1 and 10% (*w*/*w*) and the emulsifier polysorbate 80 (Tween^®^ 80) (2% *w*/*w*) were incorporated. Briefly, the required quantities of HEMA monomer were mixed with the crosslinking agent EGDMA and initiator AIBN and heated to 60 °C in sample bottles (solution A). Fatty acids were weighed in sample bottles and heated to their respective melting points, and the monomer solution (solution A) was added. These liquids were mixed thoroughly prior to being added to pre-warmed moulds consisting of two glass sheets (150 mm × 100 mm, J.E. Harrison and Co., Ltd., Glaziers, Belfast, UK) coated in a thin film of vacuum grease to attach sheets of silicone-coated release liner. A loop of medical grade silicone tubing (outer diameter 1.19 mm, internal diameter 0.63 mm, SF Medical, Hudson, MA, USA) was placed between the sheets to form the mould and the glass plates held together by bulldog clips, forming a tight seal, which prevented leakage. Solutions were added to the moulds and maintained within a fan-assisted convection oven at 60 °C for 18 h or until polymerisation occurred. Upon cooling, films were removed from moulds and washed to remove unreacted monomer and stored in deionised water until required (typically 7 days).

#### 2.2.2. Dynamic Contact Angle Analysis of Hydrogels

The dynamic advancing and receding contact angles of sample materials were analysed by the Wilhelmy plate technique using a First Ten Angstroms Dynamic Contact Analyser (DCA-100) (First Ten Angstroms, Cambridge, UK) interfaced with a personal computer. Rectangular samples (10 mm × 30 mm) of hydrated hydrogels were cut from material sections with a sharp scalpel, with accurate measurement of the perimeter of sample sections performed with a digital micrometre. Surface moisture was removed from sample sections by blotting on filter paper prior to commencing each experimental procedure. Sections were loaded into the sample holder and allowed to equilibrate prior to recording baseline force readings. On initiation of the experimental procedure, a reservoir containing liquid of known surface tension (HPLC grade water) was raised at a rate of 0.1 mm s^−1^ to zero depth of immersion to ensure that the buoyancy effect can be neglected. Dynamic contact angles were measured as the advancing contact angle on immersion of the sample into the wetting medium and the receding contact angle upon withdrawal of the previously immersed sample. A minimum of 5 replicates were performed, and the contact angles calculated using Equation (1):

(1) $$\mathrm{Cos}\;\theta \;=\frac{F}{\mathrm{ST}\;\times \;P}$$

where F refers to the force at zero depth of immersion (ZDOI), ST is the surface tension of water, P is the sample perimeter and Cos θ is the cosine of contact angle.

#### 2.2.3. Swelling Properties of Hydrogels

Samples of hydrated hydrogels were cut using a number 8 cork borer (diameter 12 mm), dried until a constant weight was achieved, weighed and then immersed in solutions of deionised water and artificial urine (pH adjusted to 6 and 9) [9] and maintained at a constant temperature of 25 °C. The composition of artificial urine was as follows:

Solution A (5 L)
  - Potassium dihydrogen orthophosphate → 0.76% *w*/*v*
  - Magnesium chloride hexahydrate → 0.36% *w*/*v*
  - Urea → 1.60% *w*/*v*
  - Deionised water ad → 100% *w*/*v*
Solution B (5 L)
  - Calcium chloride dihydrate → 0.36% *w*/*v*
  - Chicken ovalbumin → 0.02% *w*/*v*
  - Deionised water ad → 100% *w*/*v*

Solutions A and B were prepared separately, filtered through a 0.45 mm pore size filter and then sterilised by filtration through a 0.22 μm pore size filter. The solutions were combined only prior to initiation of the experimental procedure to minimise acidic precipitation of brushite (CaHPO_4_·2H_2_O), which would lead to a reduction in the concentration of the solutions for both calcium and phosphate, as previously described by the authors [9]. The pH of the resultant solution was altered to pH 6 or 9 via the addition of sodium hydroxide (2M).

At pre-specified time intervals, hydrogel samples were removed from the solution in which it was immersed, blotted dry with filter paper to remove excess surface moisture and weighed. This procedure was repeated for each sample until no further changes in the mass of each disc were observed. The swelling ratio was determined using Equation (1):

(2) $$\mathrm{Swelling}\;\mathrm{Ratio}=\frac{\mathrm{Mass}\;\mathrm{of}\;\mathrm{hydrated}\;\mathrm{sample}\;\left(g\right)\;–\;\mathrm{Mass}\;\mathrm{of}\;\mathrm{dry}\;\mathrm{sample}\;\left(g\right)}{\mathrm{Mass}\;\mathrm{of}\;\mathrm{dry}\;\mathrm{sample}\;\left(g\right)}$$

#### 2.2.4. Tensile Analysis

**Figure 1:**
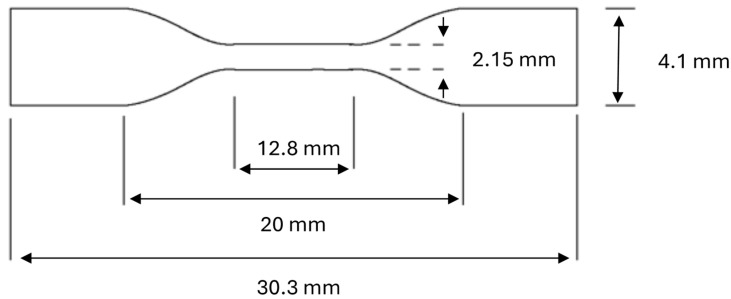
Dimensions of samples utilised for tensile analysis.

Tensile analysis of all hydrogels was carried out according to the American Standards for Testing materials guidance number D 638M using a Stable Microsystems TA-XT2 Texture analyser (Goldaming, Surrey, UK) interfaced with a personal computer. Dumb-bell-shaped samples were cut from samples hydrated and stored in deionised water, artificial urine pH 6 and pH 9 (for 14 days using a press fitted with a cutting die to dimensions as shown in Figure 1. Excess surface moisture was removed with lens tissue and sample sections fixed between upper movable grips and lower static grips utilising adhesive pads (Sellotape^®^ “Sticky fingers”™) to protect the material from excessive clamping forces. Samples were checked for alignment visually prior to commencing the experimental procedure, and the sections exposed between grips coated with light petroleum jelly minimised sample dehydration throughout testing.

During the test, the upper clamp was raised at a crosshead speed of 1.0 mm s^−1^ until sample fracture occurred (only within the narrow region of the sample). Plots of force versus time enabled calculation of the ultimate tensile strength, Young’s modulus and strain at failure using the equations below.

(3) $$\mathrm{Ultimate}\;\mathrm{Tensile}\;\mathrm{Strength}\;\left(\mathrm{MPa}\right)=\frac{\mathrm{Force}\;\mathrm{at}\;\mathrm{break}\;\left(N\right)}{\mathrm{Cross}-\sec\mathrm{tional}\;\mathrm{area}\;\mathrm{of}\;\mathrm{sample}\;\left({\mathrm{mm}}^{2}\right)}$$

(4) $$\mathrm{Strain}\;\mathrm{at}\;\mathrm{failure}\;=\frac{\mathrm{Extension}\;\mathrm{at}\;\mathrm{break}\;\left(\mathrm{mm}\right)}{\mathrm{Original}\;\mathrm{length}\;\left(\mathrm{mm}\right)}$$

(5) $${\mathrm{Young}’s\;\mathrm{modulus}}^{*}=\frac{\mathrm{Stress}}{\mathrm{Strain}}$$

#### 2.2.5. Bacterial Adherence to Hydrogels

Inoculates of *E. coli*, *S. epidermidis* and *P. mirabilis* were added to Mueller–Hinton broth and grown overnight in an orbital incubator maintained at 37 °C for overnight until stationary phase growth was attained. These bacterial cultures were then centrifuged at 3000 rpm for 10 min, and the resulting pellets re-suspended in sterile ¼ strength Ringers Solution (QSR) to OD_540_ 0.8, corresponding to an approximate concentration of 1 × 10^8^ cfu/mL. Viable counts were enumerated by the Miles and Misra drop technique onto Mueller–Hinton agar plates and incubated overnight at 37 °C [24].

To assess the adherence of bacterial species employed to test biomaterial sections, three replicate discs were placed on a hypodermic needle and placed within a sterile McCartney bottle containing minimal media. To these specimens, 20 mL of bacterial suspension was added, and the bottles were stored in an orbital incubator (37 °C, 100 rpm) for 4 h and 24 h, respectively. Samples were removed after the pre-determined time intervals and washed gently three times with 20 mL QSR solution to remove non-adhered bacterial cells. To remove adhered cells, individual sample discs in 10 mls QSR and sonicating for five minutes in an ultrasonic bath, with each tube subsequently being vortexed for 30 s, and sample discs being removed to prevent re-adherence of cells to the material. Enumeration of viable counts was performed by plating onto Mueller–Hinton agar plates (after serial dilution) and incubated overnight at 37 °C. The results are expressed as the percentage of the original inoculum adherent to the hydrogels [25].

#### 2.2.6. Resistance of Hydrogels to Encrustation

The resistance to encrustation was determined using two models, as previously reported by the authors [9]:

#### The CDC Biofilm Reactor Model

*Pr. mirabilis* (NCTC 11938) was stored on Preserver Beads (Pro-Lab Cheshire, UK) at −70 °C, subcultured to Mueller–Hinton broth (MHB) and grown overnight at 37 °C in an orbital incubator. One millilitre of this broth was then added to fresh MHB and left to grow for 4 h to early exponential phase. Samples of this broth were then centrifuged at 3000 rpm for 10 min and re-suspended in ¼ strength Ringers Solution to OD_540_ 0.8, corresponding to an approximate concentration of 1 × 10^8^ colony forming units mL^−1^. Viable counts were enumerated by the Miles and Misra drop technique onto Mueller–Hinton agar plates and incubated overnight at 37 °C [25].

The assembled (sterile) biofilm reactor system was placed in an isothermal water bath at 37 °C, and into this was added 15 mL of MHB, 10 mL inoculum of *Pr. mirabilis* and artificial urine (equal parts solutions A and B) to a final volume of 400 mL. The model was run as a batch system for 4 h before initiation of artificial urine flow (3 mL min^−1^). The hydrogel samples were immersed into the reactor system and, at pre-selected time intervals, removed and the masses of adherent calcium and magnesium salts quantified using Atomic Absorption Spectroscopy as previously reported [9].

#### The Urease Model

In this model, equal volumes of solutions A and B (300 mL) were added to the reaction vessel, the pH adjusted using sodium hydroxide, as described above, and to this, 3.84 mL of urease solution (1 unit mL^−1^) was added. The hydrogel samples were immersed in this solution. Over the duration of the study (14 d), 63.84 mL of artificial urine was removed from the reaction vessel and replaced with 60 mL of pre-warmed solutions A and B and 3.84 mL of urease solution. As before, the masses of adherent calcium and magnesium salts were determined using Atomic Absorption Spectroscopy.

#### 2.2.7. Statistical Analysis

The effect of media type (deionised water, pH 6 and pH 9) and material composition on the mechanical properties (ultimate tensile strength, % elongation at break and Young’s modulus), the swelling properties and the adherence of each microorganism were statistically determined using a two-way ANOVA. The effects of material composition and time of contact on the adherence of each were statistically examined using a two-way Analysis of Variance (ANOVA). The effect of material composition on the advancing and receding contact angles was statistically examined using a one-way Analysis of Variance (ANOVA). Similarly, the effect of material composition and storage time on encrustation (mass of both calcium and magnesium) were determined using a two-way ANOVA. Fisher’s Least Significant Difference (LSD) was employed to identify individual differences between variables. In all cases, *p* ≤ 0.05 denotes significance and, accordingly, individual *p* values are not presented.

## 3. Results and Discussion

Over the previous decades, increasing utilisation of indwelling urethral catheters within primary and secondary care settings has been reported. For example, Shackley et al. reported that the incidence of urinary catheter usage in the population was 12.7% (with 18.6% of patients using urinary catheters in hospitals, 7.0% within community and 24.4% in hospices [26]. Most recently, a report from the Centre for Disease Control [27] stated that circa 75% of all urinary tract infections are associated with urinary catheters, that catheter-associated urinary tract infections (CAUTIs) are one of the most common hospital-acquired infections and are associated with increased healthcare costs, morbidity and mortalities. Whilst not as prevalent, encrustation of urinary catheters in patients undergoing long-term catheterisation presents a major clinical problem [28]. Encrustation is clinically linked to infection, with studies showing that urease-producing microorganisms (*Proteus* species) (and the associated increase in urinary pH) is the dominant mechanism of encrustation [28,29].

As detailed previously, several strategies with varying degrees of success have been examined to reduce CAUTIs (and encrustation [30]. Given the linkage between CAUTIs and encrustation, the need to reduce microbial colonisation and biofilm formation on the catheter is clear. The inclusion of antimicrobial agents, including antibiotics, non-antibiotic antimicrobial agents and silver, within medical devices has been extensively examined; however, it has been noted that due to their low to moderate efficacy, the incidence rate of CAUTIs remains high and may result in increased antimicrobial resistance [31,32]. In this study, we describe the potential use of fatty acid-containing hydrogels as a platform for urinary catheters for improved biological performance. Fatty acids have been chosen on account of their inherent antimicrobial properties and minimal incidence of antimicrobial resistance [33].

### 3.1. Characterisation of the Bulk (Mechanical and Swelling) and Surface Properties (Contact Angle) of the Fatty Acid-Containing p(HEMA) Hydrogels

**Figure 2:**
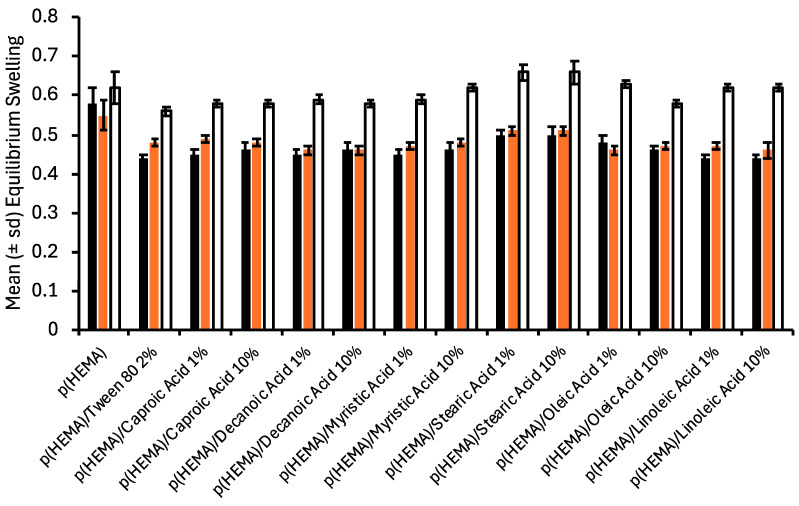
The effect of incorporation of fatty acids on the equilibrium swelling of p(HEMA) hydrogels * (black bars refer to equilibrium swelling in deionised water, red bars refer to equilibrium swelling in artificial urine pH 6, and clear bars refer to equilibrium swelling in artificial urine pH 9). * All fatty acid-containing hydrogels contained 2% Tween^®^ 80.

The bulk properties of the various hydrogels were characterised by their swelling properties (Figure 2) and mechanical properties (Table 1). Whilst individual differences were observed in the equilibrium swelling ratios of the various hydrogels, the most significant differences were associated with the fluid in which the samples were stored. Swelling of the various hydrogels in deionised water (pH 5.5) and pH 6 (artificial urine) were statistically similar and, except for p(HEMA) control, were lower than those stored at pH 9 (artificial urine). As expected, the swelling kinetics of p(HEMA) control was unaffected by the composition of the swelling fluid, as would be expected given that this polymer is unionised at these pH values. The reduced equilibrium swelling ratio of fatty acid-containing hydrogels at pH 6 may be accredited to the greater hydrophobicity of these materials. Whilst the pKa of fatty acids in the free molecular state has been reported as *circa* 4.5–6 [34,35], which can increase by several units when present in more complex structures, e.g., micelles [34,36,37]. As the concentration of the various fatty acids exceeds the critical micelle concentration, the fatty acid will be present as micelles, the higher apparent pKa of the fatty acids in this state, thereby suppressing ionisation and hence the hydrophobicity of the hydrogel. The equilibrium swelling ratios of hydrogels containing fatty acids at pH 9 increased and approximated that of p(HEMA) control. The ionisation of the fatty acids may explain this [35]; their subsequent release into the bathing solution renders the hydrogel more hydrophilic. In addition, at pH 9, high degrees of ionic repulsion between carboxylate ions reduce molecular packing, increasing the void space in materials [35].

pKa values of short-chain fatty acids are *circa* 4.5–6 [34,35,36], and pKa of unsaturated fatty acids reported as *circa* 8–10 [35,37], each dependent on the method of measurement (surface or bulk pKa) and the state of the fatty acid, e.g., molecular state, within micelles or a biological membrane.

The effects of fatty acids on the mechanical properties (ultimate tensile strength, Young’s modulus and % elongation at break) of p(HEMA) hydrogels are shown in Table 1. Again, whilst there were individual differences between the ultimate tensile strengths of the various materials, the greatest (and significant) effects of fatty acid/Tween^®^ 80 addition (particularly at the 10% *w*/*v* concentration) were observed following storage in artificial urine at pH 9, in which the ultimate tensile strengths decreased. The Young’s modulus and % elongation at break of all fatty acid-containing hydrogels (independent of the nature of the fluid in which they were stored) were significantly lower than those of p(HEMA); however, they were still within the ranges that would be suitable for clinical usage [38,39]. The compromised mechanical properties may be ascribed, at least in part, to the reduced mass of HEMA monomer required to facilitate the inclusion of Tween^®^ 80 and each fatty acid. Furthermore, incorporating fatty acid will result in increased free volume within the polymer, with increased molecular spacing, disrupting intramolecular interactions within the material structure [40].

**Figure 3:**
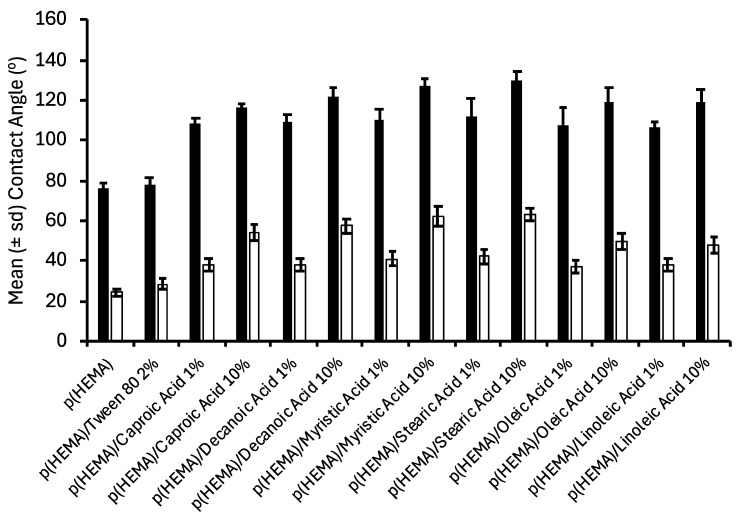
Contact angle analysis of fatty acid containing p(HEMA) biomaterials * (black bars refer to advancing contact angles, and clear bars refer to receding contact angles). * All fatty acid containing hydrogels contained 2% Tween^®^ 80.

The contact angle of biomaterials has been reported to significantly affect their biological performance. For example, Cai et al. [41] ascribed the reduced bacterial adherence to hydrogels (in part) to their greater hydrophilicity. Similarly, Chu et al. [42] reported decreased bacterial adherence to agarose-coated silicone rubber surfaces and partially accredited this to the low contact angle of these biomaterials. The advancing and receding contact angles of the various biomaterials in this study are presented in Figure 3. The advancing contact angles of the biomaterials exceeded the receding contact angles (hysteresis) due to the reorientation of polymer side chains upon immersion in water [43]. The inclusion of fatty acids increased the contact angles of p(HEMA); the effect of type of fatty acid was insignificant, but increasing fatty acid concentration significantly increased the contact angles. It may thus be inferred that there is an orientation of the various fatty acids at the surface of the biomaterials within the aqueous environment and that the mass of fatty acid at the interface increased with increasing fatty acid content.

### 3.2. Characterisation of the Resistance of Fatty Acid Containing p(HEMA) Hydrogels to Microbial (Proteus mirabilis, Escherichia coli, Staphylococcus epidermidis) Adherence

Due to the range of complications associated with urinary catheters, alteration of the characteristics of biomaterials through surface modification or incorporation of active agents to improve bacteriological performance is a purposeful intervention [5,32]. As previously detailed, strategies include the modification of the surface properties of the catheter, use of biodegradable coatings [17,25] and the inclusion of antimicrobial agents [12,14]. The efficacy of the incorporation of antimicrobial agents within medical devices has been debated [14,31,32], and, in addition, issues relating to the development of resistance have been raised [31,44]. It must be noted, however, that catheter-associated urinary tract infection (CAUTI) is a complex (multivariate) process, but poor design of antimicrobial release from the catheters will play a significant role in the performance of these catheters.

As detailed previously, fatty acids have been chosen in this study for both their inherent antimicrobial properties [20,33,35] and their ability to modify the hydrophilicity/hydrophobicity of the catheter surface, as shown in Table 2, the latter being of interest due to the initial stage of colonisation of urinary catheters requiring adherence to the catheter surface [45]. The ability of antimicrobial agents to reduce the adherence of microorganisms to epithelial cells [46] and polymeric surfaces [15,39,47,48,49] has been known for many years. However, there is a paucity of studies that have reported the ability of fatty acids to similarly interfere with microbial adherence to surfaces. Recently, Jin et al. described the ability of fatty acids to repress the formation of *E. coli* biofilms, with lauric acid showing an eight-fold reduction in the number of colony-forming units per unit area of *E.coli* within the biofilm compared with the DMSO control [21]. This study has uniquely shown that certain fatty acids, when incorporated within p(HEMA) offer reduced microbial adherence to this biomaterial after contact times of 4 h and 24 h. The type of fatty acid, but not the concentration, significantly affected microbial adherence to p(HEMA). In addition, the effect of the fatty acids on adherence was dependent on the microorganism. In general, reduced adherence of the test microorganisms was associated with caproic and decanoic acid-containing p(HEMA) hydrogels, with some anti-adherence properties associated with myristic acid containing p(HEMA) (but not against all three test microorganisms). Exemplar observed reductions in adherence were *circa* 78% (4 h adherence) and 73% (24 h adherence) observed for myristic acid against *E. coli*, 87.5% (4 h adherence) and 95% (24 h adherence) observed for caproic acid against *Pr. mirabilis* and *circa* 67% (4 h adherence) and 90% (24 h adherence) observed for decanoic acid against *S. epidermidis*. In their study to determine the inhibitory effect of fatty acids on *E. coli* biofilm growth, Jin et al. reported the ability of undecanoic acid, N-tridecanoic acid and (especially) lauric acid to reduce biofilm growth when grown in the presence of 1 mM of each of the fatty acids for 24 h [21]. This current study has successfully shown that, after contact for both 4 h and 24 h, p(HEMA) containing fatty acids (principally caproic acid, decanoic acid and myristic acid) successfully reduced the adherence of three microorganisms that are frequently associated with CAUTIs [50,51]. Unlike the study by Jin et al., this study has successfully demonstrated that certain fatty acids, when incorporated with p(HEMA) (a model hydrogel), offer an increased resistance to microbial adherence at both 4 h and 24 h. The mechanism for the observed anti-adherence effects is unclear but may be due to the antimicrobial properties of the fatty acids immediately at the interface between the hydrogel and the aqueous fluid. Given the reported importance of the use of urinary catheters and reduced urinary tract infections [52,53], a correlation analysis was performed to examine the relationship between biomaterial contact angle (a measure of the hydrophilic/hydrophobic properties) and adherence of the microorganisms to the biomaterials. No relationship was observed between the contact angles of the biomaterials and the observed resistances of the biomaterials to microbial adherence, and therefore, the contribution of this property to adherence is minimal.

As detailed previously, a significant problem associated with the use of urinary catheters is encrustation, the deposition of insoluble calcium and magnesium salts on the catheter surface, resulting in blockage of the flow of urine, damage to the urethra upon removal, problems in deflating the balloon and is frequently associated with CAUTI [8,10,13,53]. Therefore, in addition to increased resistance to microbial colonisation, biomaterials designed for urinary use should offer increased resistance to urinary encrustation. Whilst the relationship between the elevated pH of urine (due to the presence of urease-producing microorganisms on the catheter surface or metabolic diseases) and encrustation is clear [9,10,53], the relationship between the biomaterial composition and encrustation is less understood. Several strategies have been proposed to reduce/inhibit the development of encrustation on urinary devices. These include (but are not limited to) the use of biodegradable coatings (e.g., poly(caprolactone) [25], poly(urethane) and magnesium alloys [54], antimicrobial coatings [55,56], coatings that offer urease-responsive antibiotic release [57], superhydrophobic coatings [11] and strategies to acidify urine and prevent crystallisation [58]. This study presents an alternative approach to reduce encrustation, which also incorporates microbial anti-adherence properties. The resistances of the various biomaterials under investigation to encrustation were assessed using two experimental models that have been previously employed by the authors [9,10,45] and depicted in Table 3 (CDC encrustation model) and Table 4 (urease encrustation model). In the urease model, urease was directly added into the salt solution, whereas in the CDC model, urease was generated in situ through inoculation of the salt solution with a urease-producing strain of *P. mirabilis*. Whilst the masses of calcium and magnesium encrustation deposited on the various hydrogels differed depending on the method used, there was a strong correlation between the two methods concerning the comparative resistances of the hydrogels to encrustation. For both fresh samples and samples that had been stored in PBS for 30 days, the incorporation of Tween^®^ 80 and the various fatty acids significantly reduced the mass of encrusted salts on the various hydrogels. Following storage for 30 days in PBS, all fatty acid and Tween^®^ 80 containing hydrogels exhibited greater encrustation in comparison to their fresh counterparts but still less than the p(HEMA) control. The type and concentration of the fatty acids significantly affected the resultant encrustation on the hydrogels, with caproic acid and decanoic acid showing the greatest reduction in encrustation in the CDC method and additionally stearic and oleic acids in the urease method. Hydrogels containing a higher concentration of fatty acid showed greater resistance to encrustation than those with a lower concentration. The observed differences in the two encrustation methods are due to the differences in the mass of urease produced by each method, with the CDC model showing the resistance of the hydrogels in the presence of higher concentrations of urease [9]. The reductions in encrustation associated with the fatty acid containing hydrogels were both significant and marked. For example, the % reductions in encrustation of fresh samples of p(HEMA) containing 10% *w*/*w* caproic acid were *circa* 91% (each for calcium and magnesium) whereas the reductions shown by fresh samples of (HEMA) containing 10% loaded decanoic acid were *circa* 93% (calcium) and 96% (magnesium) (CDC encrustation method). This study has shown that encrustation (as assessed by each method) is lower on the more hydrophobic fatty acid containing biomaterials than on p(HEMA) alone or p(HEMA) containing Tween^®^ 80, and accordingly, surface energy may contribute to the reduced encrustation. The ability of hydrophilic coatings to reduce encrustation has been widely reported. For example, Tunney and Gorman reported the increased resistance to encrustation of a poly(vinyl pyrrolidone)-coated polyurethane when compared to the native polymer [59], whereas Jones et al. described the greater resistance of diamond-like carbon-coated polyurethane to the native polymer, the coating increasing the hydrophilicity of polyurethane [45]. Similarly, Liu et al. described a coating composed of poly(vinyl pyrrolidone) and citric acid (deposited onto polyurethane that had been pretreated with an oxygen plasma) that offered increased resistance to encrustation in an in vitro dynamic encrustation model [60]. The ability of hydrophobic surfaces to reduce encrustation has also been reported; e.g., Teng et al. examined the anti-fouling and anti-encrustation properties of superhydrophilic zwitterionic surfaces, slippery liquid-infused porous surfaces, covalently attached liquid-like surfaces, and superhydrophobic surfaces and highlighted, in particular, the success of the first two categories of biomaterial [61]. Lim et al. modified the surface of polyurethane using chemical vapour deposition and reported greater encrustation on surfaces that rendered polyurethane more hydrophilic, whereas the more hydrophobic C_2_H_2_ vapour-deposited coating showed reduced (5-fold) encrustation [62]. It may be concluded that the relationship between contact angle and encrustation is complex, and therefore, whilst the increased contact angle associated with the various fatty acid-p(HEMA) hydrogels may contribute, they are not solely responsible for the observed reductions in encrustation. Considering this and the greater reductions in encrustation on p(HEMA) containing the higher concentrations of fatty acids, it is more likely that the main effect of fatty acids on encrustation is due to both the mass of fatty acid at the interface and the interaction of this with the components of artificial urine. At the pH of artificial urine (pH 9), the fatty acids at the biomaterial–artificial urine interface will be ionised and will consequently interact with the calcium and magnesium salts of the artificial urine. Calcium and magnesium salts of fatty acids can precipitate at the interface and, in so doing, produce a temporarily greasy surface [63] that renders the surface more resistant to the development of encrustation. All fatty acids containing hydrogels additionally included Tween 80 (2% *w*/*v*), and thus, it is additionally plausible that the concurrent release of this surfactant may influence the formation kinetics and physicochemical properties of the calcium/magnesium fatty acid salts. The ability of a second surfactant to act as a wetting agent of saturated solutions of calcium salts of fatty acids has been previously reported by Ballusuwatthi et al. [64]. Whilst the observed reductions in encrustation are extremely encouraging, a further enhancement of this reduction may be possible by modifying the concentration of a second surfactant to facilitate removal of encrustation thereby cleaning the surface of the catheter. This aspect is under current investigation.

## 4. Conclusions

This study has uniquely described the design and physicochemical characterisation of p(HEMA) hydrogels containing a wide range of fatty acids (and Tween^®^ 80 added to ensure homogeneity within the hydrogels) and has characterised their in vitro resistance to microbial adherence (*E. coli*, *S. epidermidis* and *Pr. mirabilis*) and encrustation. The presence of fatty acids reduced the equilibrium swelling and mechanical properties of p(HEMA), the mechanical properties suggesting a greater suitability of the hydrogels as coatings. Incorporation of the various fatty acids into the hydrogels increased the contact angle (advancing and receding) of p(HEMA) in a concentration-dependent fashion due to the location of the fatty acids at the water–hydrogel interface. The presence of certain (but not all) fatty acids significantly reduced the adherence of *E. coli*, *Pr. mirabilis* and *S. epidermidis* to p(HEMA) hydrogels; however, these reductions were modest. Decanoic acid and myristic acid provided the greatest anti-adherence properties against the three microorganisms. No direct correlations were observed between the contact angle of the hydrogels and their resistance to microbial adherence. The resistance of the fatty acid containing hydrogels to urinary encrustation was assessed using two in vitro methods with good correlations observed between each method. Importantly, most fatty acids containing hydrogels exhibited increased resistance to encrustation in a concentration-dependent fashion, with caproic acid, decanoic acid, myristic acid and stearic acid showing the greatest resistance (in both encrustation models). It is proposed that the advantageous resistance to encrustation is due both to the formation of the calcium/magnesium fatty acid salts at the surface, which offers a greasy surface, and, in addition, the contribution of Tween^®^ 80 in facilitating the removal of the fatty acid salts from the surface of the hydrogel. The observed enhanced resistance of the hydrogels to encrustation offers opportunities for the use of such systems as platforms for coatings of urinary catheters. Such systems could be formulated to include more potent non-antibiotic, antimicrobial agents, e.g., chlorhexidine, to augment the microbial anti-adherence properties of the hydrogels and, in so doing, provide a complete solution for the reduction in CAUTI and urinary encrustation.

## Figures and Tables

**Table 1:** Mechanical properties of fatty acid containing p(HEMA) biomaterials following swelling in deionised water and artificial urine (pH 6 and 9).

Biomaterial *	Concentration (*w*/*w*)	Mean (±sd.) Ultimate Tensile Strength (MPa)			Mean (±s.d.) Young’s Modulus (MPa)			Mean (±s.d.) % Elongation at Break		
		Deionised Water	Artificial Urine pH 6	Artificial Urine pH 9	Deionised Water	Artificial Urine pH 6	Artificial Urine pH 9	Deionised Water	Artificial Urine pH 6	Artificial Urine pH 9
p(HEMA)	0	0.8 ± 0.1	0.8 ± 0.2	0.8 ± 0.0	1.5 ± 0.1	1.5 ± 0.2	1.6 ± 0.1	146.4 ± 12.1	152.8 ± 10.1	139.2 ± 9.9
p(HEMA)/Tween 80	2	0.7 ± 0.1	0.8 ± 0.0	0.8 ± 0.1	1.0 ± 0.1	0.7 ± 0.0	0.9 ± 0.1	65.9 ± 7.0	87.7 ± 4.4	74.9 ± 12.0
p(HEMA)/Caproic Acid	1	1.1 ± 0.1	0.9 ± 0.1	0.5 ± 0.0	1.4 ± 0.1	1.1 ± 0.1	0.9 ± 0.0	60.0 ± 6.3	68.2 ± 9.3	61.1 ± 4.6
p(HEMA)/Caproic Acid	10	0.8 ± 0.1	0.9 ± 0.1	0.6 ± 0.1	1.2 ± 0.1	0.9 ± 0.0	0.9 ± 0.0	47.8 ± 4.1	84.9 ± 7.9	60.8 ± 0.9
p(HEMA)/Decanoic Acid	1	1.1 ± 0.0	0.7 ± 0.1	0.7 ± 0.0	1.2 ± 0.0	0.9 ± 0.0	0.9 ± 0.0	66.6 ± 7.8	79.1 ± 8.1	66.3 ± 4.9
p(HEMA)/Decanoic Acid	10	0.6 ± 0.1	0.6 ± 0.0	0.4 ± 0.0	0.9 ± 0.1	0.9 ± 0.0	0.8 ± 0.1	68.1 ± 5.3	49.6 ± 6.4	56.5 ± 5.4
p(HEMA)/Myristic Acid	1	0.9 ± 0.0	0.8 ± 0.1	0.6 ± 0.0	1.0 ± 0.1	0.9 ± 0.0	0.9 ± 0.0	78.2 ± 2.3	73.3 ± 7.8	70.4 ± 3.3
p(HEMA)/Myristic Acid	10	0.8 ± 0.1	0.6 ± 0.1	0.6 ± 0.0	1.1 ± 0.0	0.8 ± 0.0	1.0 ± 0.1	67.9 ± 8.4	65.9 ± 4.7	50.7 ± 3.5
p(HEMA)/Stearic Acid	1	1.0 ± 0.0	0.7 ± 0.1	0.7 ± 0.0	1.1 ± 0.1	1.1 ± 0.1	1.1 ± 0.0	74.3 ± 4.9	74.3 ± 5.1	68.3 ± 5.0
p(HEMA)/Stearic Acid	10	0.9 ± 0.1	0.7 ± 0.1	0.9 ±0.1	1.3 ± 0.1	0.8 ± 0.0	1.0 ± 0.0	64.2 ± 4.8	62.1 ± 3.9	74.9 ± 5.0
p(HEMA)/Oleic Acid	1	1.0 ± 0.1	0.8 ± 0.1	0.7 ± 0.0	1.0 ± 0.1	0.9 ± 0.1	1.0 ± 0.0	77.9 ± 10.7	69.1 ± 4.9	67.0 ± 5.4
p(HEMA)/Oleic Acid	10	0.8 ± 0.1	0.8 ± 0.1	0.5 ± 0.0	1.0 ± 0.0	0.8 ± 0.0	0.8 ± 0.0	66.3 ± 4.9	55.4 ± 5.6	88.6 ± 4.8
p(HEMA)/Linoleic Acid	1	0.9 ± 0.1	0.6 ± 0.1	0.6 ± 0.1	1.0 ± 0.1	0.7 ± 0.0	0.7 ± 0.1	87.6 ± 7.1	93.0 ± 7.3	87.3 ± 3.9
p(HEMA)/Linoleic Acid	10	0.7 ± 0.1	0.6 ± 0.1	0.5 ± 0.0	0.7 ± 0.0	0.5 ± 0.0	0.6 ± 0.0	76.9 ± 1.6	73.4 ± 4.8	78.2 ± 4.4

* All fatty acid containing hydrogels contained 2% Tween^®^ 80.

**Table 2:** The adherence of *Pr. mirabilis*, *E. coli* and *S. epidermidis* to fatty acid containing p(HEMA) hydrogels.

Biomaterial Composition and Concentration (% *w*/*v*) *		Mean (±sd) Bacterial Adherence (% of Original Inoculum, *circa* 1 × 10^8^ cfu mL^−1^) to Each Biomaterial Following 4 h Contact			Mean (±sd) Bacterial Adherence (% of Original Inoculum, *circa* 1 × 10^8^ cfu mL^−1^) to Each Biomaterial Following 24 h Contact		
		*E. coli*	*Pr. mirabilis*	*S. epidermidis*	*E. coli*	*Pr. mirabilis*	*S. epidermidis*
p(HEMA)	0	0.14 ± 0.02	0.08 ± 0.02	0.03 ± 0.01	0.11 ± 0.02	0.07 ± 0.02	0.04 ± 0.01
p(HEMA)/Tween 80	2	0.11 ± 0.02	0.04 ± 0.00	0.01 ± 0.00	0.07 ± 0.02	0.04 ± 0.01	0.02 ± 0.00
p(HEMA)/Caproic Acid	1	0.05 ± 0.01	0.02 ± 0.00	0.02 ± 0.00	0.06 ± 0.01	0.02 ± 0.00	0.02 ± 0.00
p(HEMA)/Caproic Acid	10	0.06 ± 0.01	0.01 ± 0.00	0.02 ± 0.00	0.06 ± 0.01	0.004 ± 0.002	0.01 ± 0.00
p(HEMA)/Decanoic Acid	1	0.06 ± 0.02	0.03 ± 0.01	0.01 ± 0.00	0.06 ± 0.01	0.02 ± 0.00	0.01 ± 0.00
p(HEMA)/Decanoic Acid	10	0.05 ± 0.01	0.02 ± 0.00	0.01 ± 0.00	0.05 ± 0.01	0.02 ± 0.00	0.004 ± 0.001
p(HEMA)/Myristic Acid	1	0.03 ± 0.00	0.11 ± 0.03	0.01 ± 0.00	0.03 ± 0.01	0.09 ± 0.03	0.01 ± 0.00
p(HEMA)/Myristic Acid	10	0.04 ± 0.01	0.10 ± 0.03	0.01 ± 0.00	0.05 ± 0.01	0.11 ± 0.03	0.01 ± 0.00
p(HEMA)/Stearic Acid	1	0.08 ± 0.02	0.11 ± 0.03	0.01 ± 0.00	0.06 ± 0.01	0.11 ± 0.03	0.01 ± 0.00
p(HEMA)/Stearic Acid	10	0.06 ± 0.02	0.12 ± 0.03	0.01 ± 0.00	0.09 ± 0.02	0.11 ± 0.02	0.01 ± 0.00
p(HEMA)/Oleic Acid	1	0.07 ± 0.01	0.09 ± 0.03	0.01 ± 0.00	0.08 ± 0.02	0.07 ± 0.02	0.01 ± 0.00
p(HEMA)/Oleic Acid	10	0.11 ± 0.02	0.10 ± 0.02	0.01 ± 0.00	0.12 ± 0.03	0.07 ± 0.02	0.01 ± 0.00
p(HEMA)/Linoleic Acid	1	0.12 ± 0.02	0.08 ± 0.01	0.05 ± 0.01	0.11 ± 0.02	0.10 ± 0.02	0.03 ± 0.01
p(HEMA)/Linoleic Acid	10	0.11 ± 0.02	0.08 ± 0.00	0.05 ± 0.01	0.14 ± 0.02	0.10 ± 0.03	0.07 ± 0.01

* All fatty acid-containing hydrogels contained 2% Tween^®^ 80.

**Table 3:** The effect of biomaterial composition and storage in deionised water on the resistance to encrustation, determined using the CDC method.

Biomaterial Composition and Concentration (% *w*/*v*) *		Mean (±sd) of Calcium and Magnesium Deposited on Biomaterials Following Storage, Determined Using the CDC Method (24 h)			
		Fresh Samples		Samples Stored for 30 Days in PBS	
		Calcium (μg cm^−2^)	Magnesium (μg cm^−2^)	Calcium (μg cm^−2^)	Magnesium (μg cm^−2^)
p(HEMA)	0	961.1 ± 80.9	996.3 ± 79.6	991.4 ±87.6	1007.0 ± 75.3
p(HEMA)/Tween 80	2	233.3 ± 21.3	227.3 ± 19.3	381.5 ± 29.6	394.7 ± 24.8
p(HEMA)/Caproic Acid	1	134.6 ± 6.4	121.8 ± 5.9	234.6 ± 23.5	221.8 ± 16.8
p(HEMA)/Caproic Acid	10	87.3 ± 3.0	88.7 ± 1.7	121.6 ± 18.6	134.8 ± 15.2
p(HEMA)/Decanoic Acid	1	167.5 ± 8.3	143.2 ± 9.4	201.6 ± 10.3	221.2 ± 9.4
p(HEMA)/Decanoic Acid	10	69.2 ± 5.0	34.8 ± 2.5	97.6 ± 7.9	66.4 ± 8.1
p(HEMA)/Myristic Acid	1	204.8 ± 9.9	188.7 ± 9.1	297.6 ± 23.0	271.4 ± 18.8
p(HEMA)/Myristic Acid	10	101.3 ± 5.0	77.7 ± 3.8	167.1 ± 11.4	134.3 ± 16.3
p(HEMA)/Stearic Acid	1	227.6 ± 10.4	203.8 ± 9.9	239.8 ± 19.6	251.2 ± 17.6
p(HEMA)/Stearic Acid	10	167.7 ± 7.8	104.8 ± 5.4	197.6 ±14.8	167.6 ±14.6
p(HEMA)/Oleic Acid	1	227.9 ± 12.7	212.4 ± 11.4	312.2 ± 24.7	329.7 ± 25.0
p(HEMA)/Oleic Acid	10	147.3 ± 7.3	108.6 ± 5.2	257.3 ± 14.0	221.7 ± 21.2
p(HEMA)/Linoleic Acid	1	241.2 ± 16.9	241.1 ± 19.4	337.5 ± 31.3	414.2 ± 36.7
p(HEMA)/Linoleic Acid	10	271.1 ± 16.8	268.1 ± 24.5	339.6 ± 16.4	374.5 ± 21.2

* All fatty acid containing hydrogels contained 2% Tween^®^ 80.

**Table 4:** The effect of biomaterial composition and storage in deionised water on the resistance to encrustation, determined using the urease method.

Biomaterial Composition and Concentration (% *w*/*v*) *		Mean (±sd) of Calcium and Magnesium Deposited on Biomaterials Following Storage, Determined Using the Urease Method (24 h)			
		Fresh Samples		Samples Stored for 30 Days in PBS	
		Calcium (μg cm^−2^)	Magnesium (μg cm^−2^)	Calcium (μg cm^−2^)	Magnesium (μg cm^−2^)
p(HEMA)	0	347.0 ± 32.4	389.0 ± 28.9	358.7 ± 35.4	402.1 ± 31.2
p(HEMA)/Tween 80	2	137.5 ± 13.8	128.5 ± 12.9	163.8 ± 16.4	189.6 ± 19.0
p(HEMA)/Caproic Acid	1	87.6 ± 8.8	71.4 ± 7.1	127.5 ± 12.8	114.5 ± 11.5
p(HEMA)/Caproic Acid	10	71.4 ± 7.2	61.9 ± 6.2	97.6 ± 9.8	88.7 ± 8.9
p(HEMA)/Decanoic Acid	1	71.8 ± 7.4	74.6 ±7.5	83.7 ± 8.4	88.5 ± 9.9
p(HEMA)/Decanoic Acid	10	53.8 ± 5.4	52.4 ± 5.2	77.4 ± 8.4	69.4 ± 7.0
p(HEMA)/Myristic Acid	1	64.3 ± 6.4	69.6 ± 7.0	68.9 ± 6.9	78.6 ± 8.0
p(HEMA)/Myristic Acid	10	48.6 ± 4.9	46.8 ± 4.8	59.6 ± 5.1	69.8 ± 6.4
p(HEMA)/Stearic Acid	1	73.3 ± 7.5	79.7 ± 7.0	74.5 ± 6.7	81.5 ± 9.5
p(HEMA)/Stearic Acid	10	64.7 ± 6.5	67.0 ± 6.1	67.6 ± 7.1	76.4 ± 7.5
p(HEMA)/Oleic Acid	1	77.8 ± 7.8	81.0 ± 6.1	101.4 ± 8.9	120.3 ± 11.0
p(HEMA)/Oleic Acid	10	91.6 ± 8.2	69.0 ± 5.9	100.1 ± 7.3	112.2 ± 11.3
p(HEMA)/Linoleic Acid	1	103.8 ± 15.6	91.7 ± 7.1	124.7 ± 9.9	131.4 ± 12.1
p(HEMA)/Linoleic Acid	10	99.0 ± 10.9	101.0 ± 6.2	98.8 ± 10.0	117.7 ± 11.8

* All fatty acid containing hydrogels contained 2% Tween^®^ 80.

## Data Availability

Data are contained within the article.
